# Involvement of purinergic signalling in the vasomotor response to hypochlorous acid in porcine coronary artery

**DOI:** 10.1007/s11302-025-10086-7

**Published:** 2025-04-16

**Authors:** Ashwaq Baghdadi, William R. Dunn, Vera Ralevic

**Affiliations:** School of Life Sciences, Queen’s Medical Centre, University of Nottingham, Nottingham, NG7 2UH UK

**Keywords:** Adenosine receptor, ATP, Coronary artery, Hypochlorous acid, Inflammation, P2 receptor

## Abstract

Hypochlorous acid (HOCl) is generated by neutrophils during the innate immune response. ATP is released from cells by various stimuli and during inflammation but whether ATP is released by and participates in the response to HOCl is unclear. This study investigated vasomotor effects of HOCl on the porcine coronary artery (PCA) and the involvement of ATP and purine receptors. HOCl at 100 μM induced rapid and transient endothelium-dependent relaxation followed by slow and sustained endothelium-independent relaxation. Transient endothelium-dependent relaxation was induced by 500 μM HOCl, followed by endothelium-dependent contraction, then slow endothelium-independent relaxation. 8-(p-sulphophenyl)theophylline (8-SPT), an adenosine/P1 receptor antagonist, blocked rapid relaxation and contraction to HOCl but an A_2A_ receptor antagonist, ZM 241385, and an A_1_ receptor antagonist, DPCPX, had no effect. Suramin, a P2 receptor antagonist (and membrane channel inhibitor), blocked rapid relaxation (at 100 μM HOCl) and contraction to HOCl. Other antagonists for P2, P2X1, P2Y1 and P2X4 receptors (PPADS, reactive blue 2, NF449, MRS2179 and BX430) did not affect HOCl responses. Relaxation to exogenous ATP was inhibited by 8-SPT but not by suramin suggesting that suramin block of HOCl responses may involve inhibition of membrane channels and endogenous ATP release. Apyrase, which hydrolyzes nucleotides, abolished responses to HOCl, ATP and unexpectedly adenosine. Neither probenecid nor carbenoxelone (connexin and pannexin channel inhibitors) blocked responses to HOCl. Luminescent ATP assay showed that HOCl elicited ATP release from cultures of human coronary artery endothelial cells. These findings advance our understanding of inflammation by showing that HOCl evokes endothelium-dependent vasorelaxation and contraction in coronary arteries which may involve P1 receptors implicating endogenous adenosine, possibly generated from rapid metabolism of ATP released by HOCl.

## Introduction

Inflammation is a protective immune response triggered by factors such as pathogens, injury, toxic substances and chronic diseases. When tissue is affected by any of these factors, leukocytes undergo chemotaxis toward the site of inflammation where they are further activated to initiate an innate inflammatory response which involves the production of reactive oxygen species (ROS) [1, 2]. Myeloperoxidase (MPO), a haemoprotein enzyme that is present mainly in neutrophils, can be released to generate HOCl, a ROS that acts as a microbicidal agent able to kill most pathogens but which is also pro-inflammatory and can cause tissue damage at sites of inflammation [3–6]. Elevated MPO levels have been associated with coronary heart disease [7–9], but relatively little is known about the coronary vascular actions of HOCl.

ATP is present at high concentrations inside cells and damage to cells caused by inflammation may lead to its release to the extracellular environment by necrosis, vesicular exocytosis and pannexin and connexin hemichannels [10, 11]. Mechanical stimuli such as osmotic and shear stress, and changes in pH can also trigger the release of ATP from cells, including endothelial cells, smooth muscle cells, cardiomyocytes, and erythrocytes, through vesicular exocytosis and connexin and pannexin channels [12–16]. ATP is also released during inflammation from inflammatory cells and has roles in inflammatory cell recruitment, activation and secretion of pro-inflammatory cytokines via activation of P2 receptors [11]. When inflammation occurs, the generation and release of adenosine is also greatly enhanced [17, 18]. It is not known whether the inflammatory mediator HOCl evokes cellular purine release.

Roles of extracellular ATP in blood vessels include regulation of vascular contractility via P2X and P2Y receptors [10, 19, 20]. Specific roles include sympathetic neurogenic vasoconstriction via smooth muscle P2X1 receptors [21, 22] and shear stress-evoked vasodilatation involving endothelial P2Y_1_, P2Y_2_ and P2X4 receptors [23–25]. Extracellular ATP is rapidly hydrolysed by E-NTPDases to ADP and AMP, which is then metabolised to adenosine by 5’-ectonucleotidase [26, 27]. Adenosine/P1 receptors also regulate vascular contractility [28–30].

At sites of inflammation infiltrating neutrophils can lead to the generation of high concentrations of HOCl, estimated to be in the millimolar range [31]. Whilst prolonged exposure to HOCl impairs endothelium-dependent relaxation [32–36], little is known about its direct vasomotor effects, which are of interest in understanding the early events and mechanisms involved in the MPO/HOCl-induced inflammatory response. In this study, PCA were used because porcine cardiovascular systems are anatomically and functionally similar to those of humans and because coronary arteries are the site of major pathological events in humans, such as coronary artery disease [37]. This study aimed to gain a better understanding of vascular inflammation and possible purine receptor involvement in the cardiovascular actions of HOCl. Our hypothesis was that HOCl causes changes in blood vessel contractility which are due to the actions of extracellular ATP released from blood vessels by HOCl, which can be measured from the involvement of purine receptors. We aimed to provide a better understanding of the early vascular events in inflammation involving HOCl and the underlying mechanisms.

## Methods

### Preparing PCA rings

Hearts were isolated from male and female pigs (large white hybrid pigs, 4–6 months old, weighing ~ 50 kg). The hearts were delivered to the laboratory in an ice-cold Krebs–Henseleit solution from a local abattoir. The Krebs–Henseleit solution contained 118 mM NaCl, 4.8 mM KCl, 1.1 mM MgSO_4_, 25 mM NaHCO_3_, 1.2 mM KH_2_PO_4_, 12 mM D-glucose, and 1.25 mM CaCl_2_ and was gassed with 5% CO_2_ and 95% O_2_. A crude dissection was carried out to isolate the proximal part of the coronary artery (diameter 4.06 ± 0.73 mm), which was then placed in pre-gassed Krebs–Henseleit solution and refrigerated overnight at 4 °C. Fine dissection was carried out the following day to remove connective tissue. The coronary arteries were then cut to segments of approximately 5 mm in length and suspended in a multichannel organ bath. Organ baths were filled with Krebs–Henseleit solution that was gassed constantly with 5% CO_2_ and 95% O_2_ and sustained at 37 °C.

### Vasomotor responses of porcine isolated coronary arteries

Each artery segment was connected via a thread to an isometric force transducer (ADInstruments). A Quad Bridge amplifier unit was used to connect the PowerLab recording system (ADInstruments, Oxfordshire, UK) to the transducers. Any change in tension was measured using GraphPad (Version 9, GraphPad Software, Inc., San Diego, CA, USA).

Coronary artery rings were tensioned to ~ 10 g and left for approximately 30–40 min to equilibrate [38]. The viability of the arteries was assessed using 60 mM potassium chloride (KCl), which was also used to determine the level to which vessels should be pre-contracted. When a stable contraction to KCl was achieved after approximately 6 min, the tissue was washed and left for 30 to 45 min to allow for its return to baseline tension, then the second KCl addition was made followed by washout and return to baseline tension. Thereafter, U46619 (9,11-dideoxy-11α,9α-epoxymethanoprostaglandin F2α) (3–30 nM), a thromboxane A_2_ analogue, was used to contract vessels to ∼50%–60% of the second KCl response.

### Experimental protocols

#### General protocol of HOCl, ATP and adenosine response

After inducing tone with U46619, the segments were allowed to equilibrate for 20 min in order for tone to stabilise. Thereafter, the relevant test compounds (HOCl: 100 and 500 μM, ATP: 100 μM, and adenosine: 30 μM) were added and the responses investigated over 60 min of incubation. Adjacent segments from the same coronary artery were used as controls.

In some experiments, the endothelium was removed by gently rubbing the innermost surface of the artery segments on Krebs–Henseleit wet tissue paper with forceps [39]. Substance P (100 nM) confirmed that this methodology was effective. Endothelium-denuded arteries responded to substance P with less than 10% of the contraction induced by U46619 [40].

#### Protocol of inhibitors and antagonists

These compounds were added immediately after the second KCl wash and were present for the duration of the experiment. During that period, U46619 was added to contract the vessels, and the responses to HOCl and ATP, and in some experiments adenosine, of the vessels were measured. The inhibitors and antagonists and the concentrations at which they were used are shown in Table 1.

**Table 1:** Inhibitors and antagonists used

Group	Inhibitor or antagonist	Concentration (Bath)	Reference
**P1 receptor antagonists**			
**Adenosine receptor antagonist**	8-(p-sulphophenyl)theophylline (8-SPT)	100 μM	[41]
**A**_**1**_** receptor antagonist**	8-cyclopentyl-1,3-dipropylxanthine (DPCPX)	10 nM	[42]
**A**_**2A**_** receptor antagonist**	4-(−2-[7-amino-2-{2-furyl}{1,2,4}triazolo{2,3-a} {1,3,5}triazin-5-yl-amino]ethyl)phenol (ZM 241358)	1 μM	[43]
**P2 receptor antagonists**			
**Non-selective P2 antagonist**	suramin	100 μM	[44]
	Pyridoxal-5′-phosphate-6-azo-phenyl-2,4-disulfonate (PPADS)	10 µM	[45]
**P2Y**_**1**_** receptor antagonist**	2'-Deoxy-*N*^6^-methyladenosine 3',5'-bisphosphate tetrasodium salt (MRS2179)	10 µM	[38]
**P2X1 receptor antagonist**	4,4′,4″,4‴-(carbonylbis(imino-5,1,3-benzenetriylbis(carbonylimino))) tetrakis-benzene-1,3-disulfonic acid and (NF449)	10 µM	[44]
**P2X1 receptor desensitising agent**	α,β-methylene ATP	10 µM	[44]
**P2X4 antagonist**	(1-(2,6-dibromo-4-isopropyl-phenyl)−3-(3-pyridyl) urea (BX430)	10 µM	[46]
**Nucleotides hydrolysis**			
	Apyrase	10 and 100 units/ml	[47, 48]
**Ecto-ATPase inhibitor**			
	6-N,N-diethyl-d-β-γ-dibromomethylene adenosine triphosphate (ARL67156)	100 μM	[39]
**Pannexin 1 and connexin channels blocker**			
**Pannexin 1 channel blocker**	Probenecid	1 mM	[49]
**Pannexin 1 and connexin channels blocker**	Carbenoxolone	100 μM	[50]

#### Human coronary artery endothelial cells (HCAECs) culture

HCAECs (ScienCell Research Laboratories, CA, USA) (passage 6–9) were cultured in endothelial cell medium (ScienCell Research Laboratories) consisting of 500 ml basal medium, 25 ml foetal bovine serum (Cat. #0025), 5 ml penicillin/streptomycin solution (Cat. #0503), and 5 ml endothelial cell growth supplement (Cat. #1052). In a T-75 flask, the cells were grown in an incubator at 37 °C in 5% CO_2_/95% air until they reached 80%–90% confluency and then passaged every 3 to 4 days. Dulbecco's phosphate-buffered saline (Ca^2+^, Mg^2+^, and phenol red-free, Cat. #0303) was used to remove any residual bovine serum and then cells were detached using 3 ml of 0.05% trypsin/ethylenediaminetetraacetic acid (EDTA) (0.05% trypsin and 0.5 mM EDTA) solution added for 3–5 min. The cell suspension was diluted in culture medium and centrifuged for 5 min at 1000 RPM. Following suspension of the cell pellet in a suitable volume of fresh culture medium, the cells were aliquoted into a flask containing 15 ml of medium. The cells were counted using a TC20 automated cell counter (Bio-Rad, Hercules, CA, USA) and then seeded in a 6-well cell culture plate at a density of 2.5 × 10^5^ cells/well.

#### ATP assay

A luminescent ATP assay kit (Abcam, Cat#ab113849) was used to assess the effect of 100 μM HOCl on ATP levels in the bathing solution of HCAECs. After 24 h of being seeded, cells (incubated in 1 ml of medium) were treated with HOCl (100 μM) or the same volume of endothelial cell medium (10 μL, vehicle control) and then incubated for 1 and 5 min. Bathing solution (100 μL) from each well was aliquoted in a 96-well plate and then assayed for ATP according to the manufacturers instructions and using a luminescence plate reader to determine the presence and quantity of the ATP in the samples.

### Data analysis

Data are expressed as the mean ± standard error of the mean (SEM) of at least six independent experiments where *n* is the number of animals. GraphPad Prism (version 9, GraphPad Software, La Jolla, CA, USA) was used to generate plots of response against time. The contraction and relaxation responses to HOCl and ATP were expressed as a percentage (%) of the contraction induced by U46619 (measured as the increase above baseline). For the HOCl and ATP responses, tension was recorded at 1-min intervals for the first 10 min and then at 5 min intervals for a further 50 min (60 min in total). To compare differences between two groups, a two-tailed, unpaired Student's *t*-test was used. Two-way ANOVA was performed to assess differences between three or more groups. Statistical significance was defined as *p* values less than 0.05. The rapid relaxation and contractile responses were measured at the peak response. The prolonged relaxation was measured at 60 min. Responses were expressed as a percentage of U46619-induced tone.

### Drugs and chemicals

Except for BX430, ZM 241385 and DPCPX, which were obtained from Tocris-Cookson (Bristol, UK), all drugs were purchased from Sigma-Aldrich (Poole, UK). Sodium hypochlorite was from Sigma. BX430, ZM 241385 and DPCPX were dissolved in ethanol. Stock U46619 solutions were dissolved to 10 mM in ethanol, and further dilutions were made using distilled water. Probenecid was dissolved in NaOH (1 M). All further stock solutions and dilutions were made using distilled water. All stock solutions were frozen at − 20 °C.

## Results

### Multiphasic response to HOCl and endothelium involvement

**Fig. 1:**
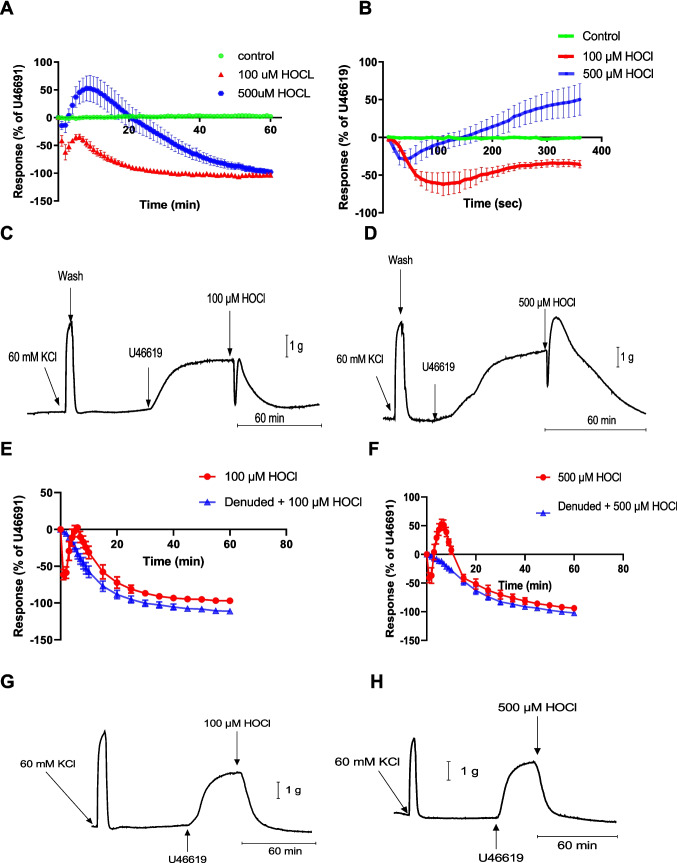
Responses of porcine coronary arteries induced by HOCl at 100 and 500 μM and the effect of endothelium removal. (**A**) responses measured for 60 min at 1 min intervals; (B) expanded time course of the first 6 min measured at 10 s intervals (360 s) to show the initial rapid relaxation; (**C–D**) representative traces for: (**C**) 100 μM HOCl and (**D**) 500 μM HOCl. Porcine coronary artery responses evoked by (**E**) 100 μM HOCl and (**F**) 500 μM HOCl in endothelium-intact and denuded vessels (*n* = 6). (**G–H**) representative trace for: (**G**) 100 μM HOCl and (**H**) 500 μM HOCl in endothelium-denuded vessels. Representative traces C,D,G and H show the second of two contractions to KCl (60 mM) which was used to set the level of precontraction. Artery segments were pre-contracted with U46619. Data are means ± SEM. Control (*n* = 6), 100 μM HOCl (*n* = 7), and 500 μM HOCl (*n* = 7)

To determine the effect of HOCl on the PCA, different concentrations (100 and 500 μM HOCl) were added to the PCA rings pre-contracted with U46619. At 100 μM, HOCl induced a rapid and transient initial relaxation (62.45 ± 13.87%) which lasted 2–4 min and then returned to baseline after 5–6 min; after that, further relaxation was observed for the remaining 60 min (103.63 ± 3.94%) (*n* = 7) (Fig. 1A). A rapid and transient relaxation response (28.77 ± 11.48%) was also induced by 500 μM HOCl, lasting about 40 s, followed by contraction which peaked at between 6 and 10 min (52.39 ± 23.27%), and then slow relaxation was observed (97.80 ± 4.20%) (*n* = 7) (Fig. 1A). Figure 1B is a plot of the first six minutes to show more clearly the initial rapid and transient responses to 100 and 500 μM HOCl.

The involvement of the endothelium in the responses to HOCl was then investigated. In endothelium-denuded PCA, the transient relaxations observed in PCA with intact endothelium to 100 μM and 500 μM HOCl (70 ± 4.5%, *n* = 6 and 57.5 ± 5.5%, *n* = 6, respectively) were abolished (Fig. 1E-H). The contraction at 500 μM HOCl in endothelium-intact arteries (54.7 ± 8%, *n* = 6) was also abolished in endothelium-denuded arteries (Fig. 1F, H). The relaxation to 100 μM HOCl at 60 min was significantly larger in the PCA without endothelium (111 ± 3.2%, *n* = 6) compared to endothelium-intact PCA (97 ± 1.5%, *n* = 6) (*p* = 0.002) (Fig. 1E).

The levels of U46119 pre-contraction (% of KCl) achieved in the vessels without endothelium in the 100 and 500 µM HOCl groups were 55.4 ± 4.5% and 60 ± 2%, respectively (both *n* = 6). Moreover, with the endothelium intact, the contraction levels with U46619 were 60 ± 5% and 59 ± 3.3% for 100 and 500 µM HOCl, respectively (both *n* = 6). There was no significant difference in tone between the vessels with and without endothelium for either the 100 µM HOCl experimental group (*p* = 0*.*47) or the 500 µM HOCl experimental group (*p* = 0*.*47). The concentration of U46619 used to pre-contract the coronary arteries was significantly lower in the vessels without endothelium (4.3 ± 0.4 nM) than in those with intact endothelium (7.5 ± 0.9 nM, *n* = 6, and 6) (*p* < 0.007).

### Purine receptor involvement in HOCl vasomotor responses

Comprehensive data for the effects of antagonists and inhibitors on the rapid and slow relaxation response to 100 μM HOCl described in this section are in Tables 2 and 3. Comprehensive data for the effects of antagonists and inhibitors on the rapid relaxation, contraction and slow relaxation to 500 μM HOCl are in Tables 4, 5, and 6.

**Table 2:** Effect of antagonists and inhibitors on rapid relaxation to 100 μM HOCl

Antagonist	Concentration	Control	Treatment	*n*	*p*
8-SPT (adenosine receptor antagonist)	100 μM	72.6 ± 4.6%	2.9 ± 1.2%	6	< 0.0001
DPCPX (A_1_ receptor antagonist)	100 nM	56.8 ± 9.3%	61.1 ± 6.3%	7	0.7
ZM 241385 (A_2A_ receptor antagonist)	1 µM	64.1 ± 3.5%	56.6 ± 2.6%	6	0.12
Suramin (non-selective P2 receptor antagonist)	100 μM	57.7 ± 10.0%	2.8 ± 1.8%	6	0.0003
PPADS (non-selective P2 receptor antagonist)	10 μM	76.6 ± 5.0%	67.8 ± 4.6%	7	0.97
MRS2179 (P2Y_1_ receptor antagonist)	10 μM	80.1 ± 6.2%	75.3 ± 6.6%	9	0.61
Reactive blue 2 (P2Y receptor antagonist)	30 μM	74.5 ± 6.5%	66.4 ± 3.2%	6	0.29
NF449 (P2X1 receptor antagonist)	10 μM	84.5 ± 5.4%	85.0 ± 3.7%	6	0.93
α,β-methylene ATP (P2X1 receptor desensitising agent)	10 μM	64.2 ± 8.1%	67.5 ± 8.0%	6	0.77
BX430 (P2X4 receptor antagonist)	10 μM	74.8 ± 6.5%	66.4 ± 3.2%	6	0.29
Apyrase (hydrolyses nucleotides)	100 units/ml	58.7 ± 5.0%	4.6 ± 1.3%	6	< 0.0001
ARL67156 (ecto-ATPase inhibitor)	100 μM	41.0 ± 9.2%	46.8 ± 9.1%	6	0.66
Probenecid (pannexin 1 channel blocker)	1 mM	69.7 ± 7.1%	78.5 ± 2.1%	7	0.25
Carbenoxolone (pannexin 1 and connexin channels blocker)	100 μM	60.6 ± 3.6%	62.1 ± 9.6%	6	0.88

**Table 3:** Effect of antagonist and inhibitors on slow relaxation to 100 μM HOCl

Antagonist	Concentration	Control	Treatment	*n*	*p*
8-SPT (adenosine receptor antagonist)	100 μM	94.5 ± 5.5%	89.0 ± 6.3%	6	0.53
DPCPX (A_1_ receptor antagonist)	100 nM	100.4 ± 2.6%	93.0 ± 2.5%	7	0.06
ZM 241385 (A_2A_ receptor antagonist)	1 µM	102.5 ± 1.9%	99.7 ± 2.8%	6	0.43
Suramin (non-selective P2 receptor antagonist)	100 μM	90.7 ± 6.3%	102.6 ± 1.7%	6	0.1
PPADS (non-selective P2 receptor antagonist)	10 μM	97.9 ± 0.9%	97.7 ± 1.2%	7	0.93
MRS2179 (P2Y_1_ receptor antagonist)	10 μM	103.36 ± 4.3%	95.3 ± 2.5%	9	0.12
Reactive blue 2(P2Y receptor antagonist)	30 μM	96.3 ± 1.3%	98.9 ± 2.4%	6	0.36
NF449 (P2X1 receptor antagonist)	10 μM	97.1 ± 1.0%	88.4 ± 5.0%	6	0.12
α,β-methylene ATP (P2X1 receptor desensitising agent)	10 μM	89.4 ± 5.6%	91.5 ± 8.8%	6	0.84
BX430 (P2X4 receptor antagonist)	10 μM	96.3 ± 3.1%	98.9 ± 2.4%	6	0.36
Apyrase (hydrolyses nucleotides)	100 units/ml	104.5 ± 9.7%	10.8 ± 3.4%	6	< 0.0001
ARL67156 (ecto-ATPase inhibitor)	100 μM	93.5 ± 1.6%	91.5 ± 2.0%	6	0.48
Probenecid (pannexin 1 channel blocker)	1 mM	94.7 ± 3.9%	105.8 ± 1.0%	7	0.01
Carbenoxolone (pannexin 1 and connexin channels blocker)	100 μM	98.8 ± 1.6%	114.1 ± 2.1%	6	0.0002

**Table 4:** Effect of antagonists and inhibitors on rapid relaxation to 500 μM HOCl

Antagonist	Concentration	Control	Treatment	*n*	*p*
8-SPT (adenosine receptor antagonist)	100 μM	42.7 ± 4.6%	Blocked	6	< 0.0001
DPCPX (A_1_ receptor antagonist)	100 nM	29.6 ± 2.8%	28.0 ± 3.3%	6	0.71
ZM 241385 (A_2A_ receptor antagonist)	1 µM	37.2 ± 5.1%	40.5 ± 5.4%	6	0.66
Suramin (non-selective P2 receptor antagonist)	100 μM	50.5 ± 5.9%	50.2 ± 8.2%	7	0.97
PPADS (non-selective P2 receptor antagonist)	10 μM	49.0 ± 7.1%	45.6 ± 5.7%	6	0.71
MRS2179 (P2Y_1_ receptor antagonist)	10 μM	61.5 ± 8.4%	58.0 ± 9.1%	6	0.78
Reactive blue 2(P2Y receptor antagonist)	30 μM	44.2 ± 5.5%	45.8 ± 4.2%	6	0.83
NF449 (P2X1 receptor antagonist)	10 μM	40.5 ± 7.2%	63.0 ± 3.3%	8	0.057
α,β-methylene ATP (P2X1 receptor desensitising agent)	10 μM	56.4 ± 9.7%	59.6 ± 10.7%	6	0.82
BX430 (P2X4 receptor antagonist)	10 μM	44.2 ± 5.5%	45.8 ± 4.2%	6	0.83
Apyrase (hydrolyses nucleotides)	100 units/ml	48.4 ± 5.1%	Blocked	6	< 0.0001
ARL67156 (ecto-ATPase inhibitor)	100 μM	29.6 ± 2.8%	28.0 ± 3.3%	6	0.71
Probenecid (pannexin 1 channel blocker)	1 mM	52.4 ± 10.8%	47.1 ± 8.1%	6	0.7
Carbenoxolone (pannexin 1 and connexin channels blocker)	100 μM	51.6 ± 5.2%	41.2 ± 6.1%	6	0.22

**Table 5:** Effect of antagonists and inhibitors on contraction to 500 μM HOCl

Antagonist	Concentration	Control	Treatment	*n*	*p*
8-SPT (adenosine receptor antagonist)	100 μM	57.3 ± 5.9%	Blocked	6	< 0.0001
DPCPX (A_1_ receptor antagonist)	100 nM	41.0 ± 11.5%	43.4 ± 14.0%	6	0.89
ZM 241385 (A_2A_ receptor antagonist)	1 µM	41.9 ± 7.7%	59.9 ± 8.1%	6	0.13
Suramin (non-selective P2 receptor antagonist)	100 μM	64.3 ± 9.1%	Blocked	7	< 0.0001
PPADS (non-selective P2 receptor antagonist)	10 μM	44.9 ± 12.0%	56.3 ± 14.7%	6	0.56
MRS2179 (P2Y_1_ receptor antagonist)	10 μM	20.2 ± 6.4%	27.9 ± 13.2%	6	0.61
Reactive blue 2(P2Y receptor antagonist)	30 μM	58.9 ± 5.6%	65.8 ± 3.8%	6	0.33
NF449 (P2X1 receptor antagonist)	10 μM	94.6 ± 9.1%	82.9 ± 15.1%	8	0.49
α,β-methylene ATP (P2X1 receptor desensitising agent)	10 μM	55.2 ± 13.7%	47.1 ± 11.5%	6	0.65
Apyrase (hydrolyses nucleotides)	100 units/ml	24.9 ± 4.6%	10.7 ± 2.8%	6	< 0.02
ARL67156 (ecto-ATPase inhibitor)	100 μM	41.0 ± 11.5%	34.5 ± 15.2%	6	0.74
BX430 (P2X4 receptor antagonist)	10 μM	58.9 ± 5.6%	64.2 ± 4%	6	0.44
Probenecid (pannexin 1 channel blocker)	1 mM	29.8 ± 12.1%	47.1 ± 19.1%	6	0.04
Carbenoxolone (pannexin 1 and connexin channels blocker)	100 μM	25.6 ± 5.5%	1.7 ± 7.4%	6	0.03

**Table 6:** Effect of antagonists and inhibitors on slow relaxation to 500 μM HOCl

Antagonist	Concentration	Control	Treatment	*n*	*p*
8-SPT (adenosine receptor antagonist)	100 μM	89.9 ± 4.4%	89.0 ± 6.3%	6	0.91
DPCPX (A_1_ receptor antagonist)	100 nM	81.8 ± 4.0%	85.7 ± 4.0%	6	0.51
ZM 241385 (A_2A_ receptor antagonist)	1 µM	94.1 ± 3.4%	99.1 ± 1.3%	6	0.2
Suramin (non-selective P2 receptor antagonist)	100 μM	79.9 ± 7.9%	97.1 ± 4.4%	7	0.08
PPADS (non-selective P2 receptor antagonist)	10 μM	86.4 ± 3.5%	81.9 ± 6.3%	6	0.54
MRS2179 (P2Y_1_ receptor antagonist)	10 μM	88.8 ± 5.0%	88.1 ± 2.8%	6	0.90
Reactive blue 2(P2Y receptor antagonist)	30 μM	92.7 ± 1.5%	98.0 ± 2.5%	6	0.12
NF449 (P2X1 receptor antagonist)	10 μM	92.1 ± 5.1%	91.6 ± 2.4%	8	0.94
α,β-methylene ATP (P2X1 receptor desensitising agent)	10 μM	79.7 ± 4.6%	89.0 ± 3.7%	6	0.15
Apyrase (hydrolyses nucleotides)	100 units/ml	81.8 ± 4.0%	85.7 ± 4.0%	6	0.51
ARL67156 (ecto-ATPase inhibitor)	100 μM	96.5 ± 6.2%	Blocked	6	< 0.0001
BX430 (P2X4 receptor antagonist)	10 μM	89.7 ± 2.8%	98.0 ± 2.5%	6	0.05
Probenecid (pannexin 1 channel blocker)	1 mM	82.1 ± 5.4%	104.5 ± 1.7%	6	0.002
Carbenoxolone (pannexin 1 and connexin channels blocker)	100 μM	89.3 ± 2.3%	104.4 ± 1.3	6	0.002

#### Effect of 8-(p-sulphophenyl)theophylline (adenosine receptor antagonist) and suramin (non-selective P2 receptor antagonist)

**Fig. 2:**
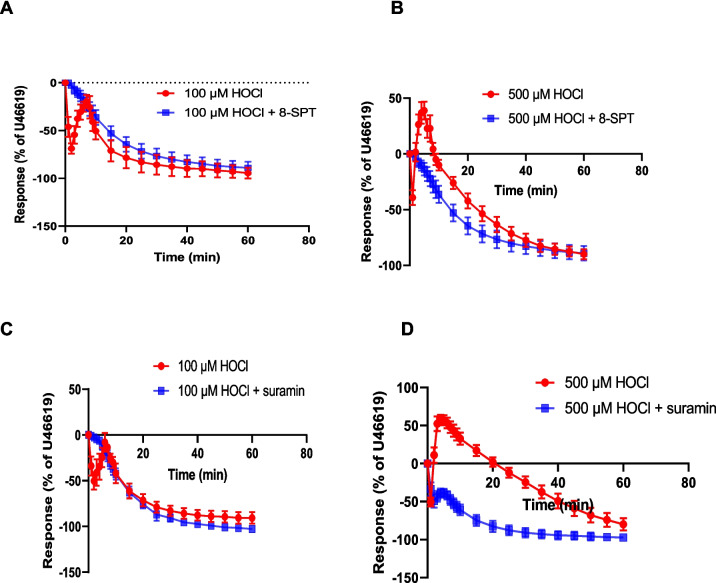
Inhibitory effects of 8-SPT and suramin on responses to HOCl. Porcine coronary artery responses produced by (**A**) 100 μM HOCl (*n* = 6) and (B) 500 μM HOCl (*n* = 6) in the absence or presence of 8-SPT (100 μM). Responses produced by (**C**) 100 μM HOCl (*n* = 6) and (**D**) 500 μM HOCl (*n* = 7) in the absence or presence of suramin (100 μM) (*n* = 6). Artery segments were pre-contracted with U46619. Data are means ± SEM

The possible involvement of purine receptors in the response to HOCl was then investigated using 8-SPT, an adenosine/P1 receptor antagonist, and suramin, a non-selective P2 receptor antagonist which can have other effects including membrane channel inhibition [12, 51]. 8-SPT (100 μM) blocked the rapid relaxation response induced by 100 μM HOCl (72.6 ± 4.6%, *n* = 6) (Fig. 2A). At a concentration of 500 μM HOCl, the transient rapid relaxation that was observed in control PCA (42.7 ± 4.3%, *n* = 6) was also blocked when 8-SPT was added (Fig. 2B). The contraction to 500 μM HOCl (57.3 ± 5.9%, *n* = 6) was blocked too in the presence of 8-SPT (Fig. 2B). These results show an involvement of adenosine receptors in the rapid relaxation and contraction to HOCl.

Suramin (100 µM) blocked the rapid relaxation observed in response to 100 μM HOCl (Fig. 2C). In contrast, the rapid relaxation observed in response to 500 μM HOCl (50.5 ± 5.9%, *n* = 7) was evident in the presence of suramin (50.2 ± 8.2%, *n* = 7) at 40 s (*p* = 0*.*97) (Fig. 2D). The contraction observed in control tissues in response to 500 μM HOCl (64.3 ± 9.1%, *n* = 7) was blocked by suramin (Fig. 2D). These data suggest a possible involvement of P2 receptors in the response to HOCl so the next experiments used other selective and non-selective P2 receptor antagonists with an aim of identifying the P2 receptor subtypes involved.

#### Effect of ZM 241385 (A_2A_ receptor antagonist) and DPCPX (A_1_ receptor antagonist) on HOCl response

The transient relaxation of PCA segments evoked by 100 μM HOCl was not different in the presence and absence of ZM 241385 (1 µM) (Table 2). Additionally, the slow relaxation at 60 min was similar in PCA with ZM 241385 compared to the control (Table 3). At 500 μM HOCl, the transient relaxation, contraction, and slow relaxation in the presence of ZM 241385 were similar to responses produced in control arteries (Tables 4, 5, and 6).

At 100 μM HOCl, DPCPX (100 nM) did not alter the transient relaxation response of HOCl compared to that in the absence of DPCPX (Table 2). Likewise, the slow relaxation response at 60 min in the absence or presence of DPCPX was similar (Table 3). With 500 μM HOCl, the rapid relaxation, subsequent contraction and the later relaxation response were also similar in the absence and presence of DPCPX (Tables 4, 5, and 6).

#### Effect of PPADS (P2 receptor antagonist), MRS2179 (P2Y_1_ receptor antagonist), reactive blue 2 (non-selective P2Y receptor antagonist), NF449 (P2X1 receptor antagonist), α,β-methylene ATP (P2X1 receptor desensitising agent), and BX430 (P2X4 receptor antagonist)

**Fig. 3:**
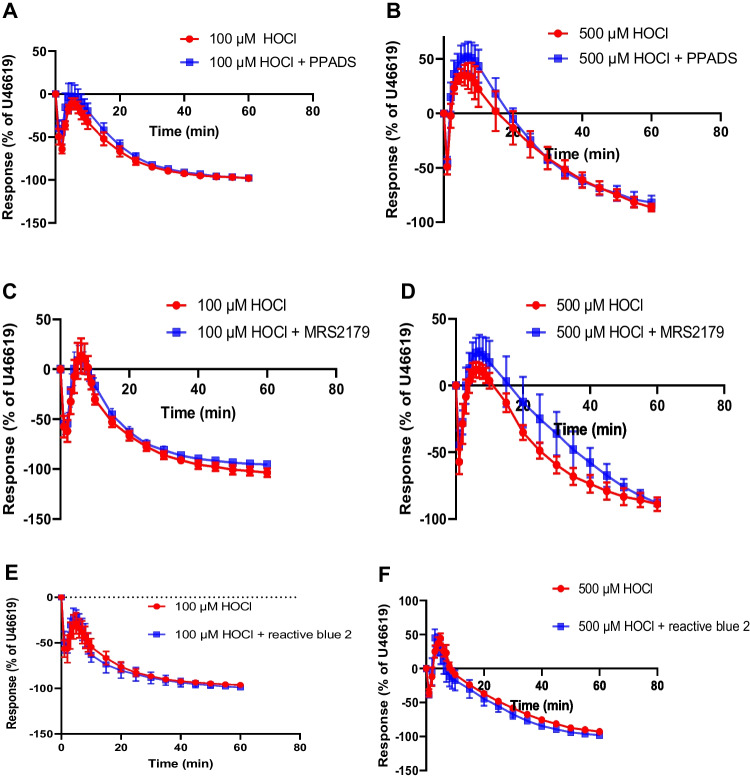

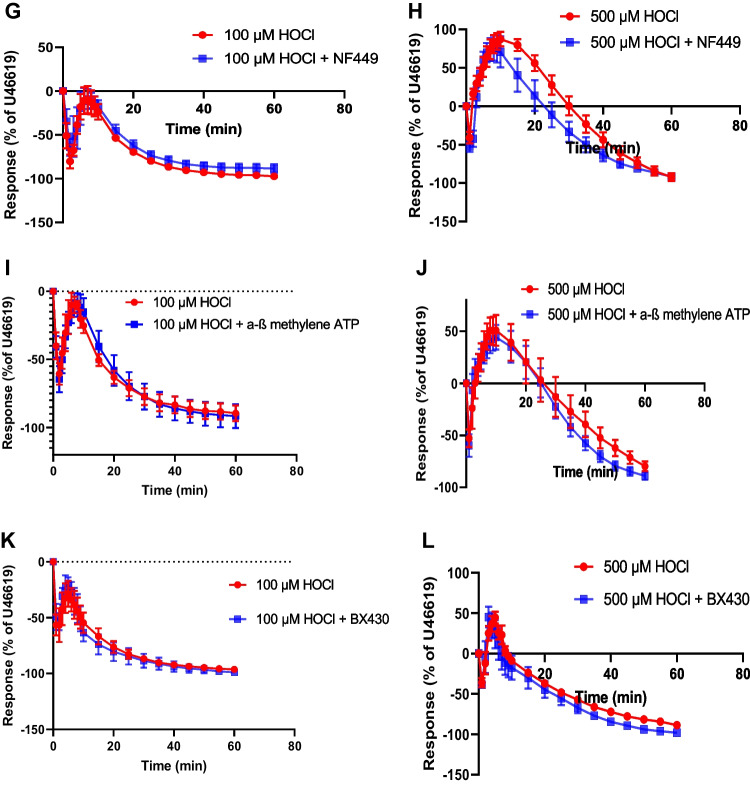
Effect of PPADS (P2 antagonist), MRS2179 (P2Y_1_ antagonist), reactive blue 2 (P2Y antagonist), NF449 (P2X1 antagonist), α,β-methylene ATP (P2X1 desensitizer) and BX430 (P2X4 antagonist) on HOCl responses. Porcine coronary artery responses produced by 100 μM HOCl in the absence or presence of: (**A**) PPADS (10 μM) (*n* = 7) (**C**) MRS 2179 (10 μM) (*n* = 9), (**E**) reactive blue 2 (30 μM) (*n* = 6), (**G**) NF449 (10 μM) (*n* = 6), (**I**) α,β-methylene ATP (10 μM) (*n* = 6) and (**K**) BX430 (10 μM) (*n* = 6). Responses produced by 500 μM HOCl in the absence or presence of: (**B**) PPADS (10 μM) (*n* = 6), (**D**) MRS 2179 (10 μM) (*n* = 6), (**F**) reactive blue 2 (30 μM) (*n* = 6), (**H**) NF449 (10 μM) (*n* = 8), (**J**) α,β-methylene ATP (10 μM) (*n* = 6) and (**L**) BX430 (10 μM) (*n* = 6). Data are means ± SEM

PPADS (10 μM), a non-selective P2 receptor antagonist, did not alter the effect of 100 μM HOCl (Fig. 3A). In addition, there was no difference between responses to 500 μM HOCl in the absence and presence of PPADS (Fig. 3B).

The effect of 100 μM HOCl on the PCA segments was similar in the presence and absence of MRS 2179 (10 µM), a P2Y_1_ receptor antagonist (Fig. 3C). At 500 μM HOCl, the transient relaxation, contraction, and slow relaxation in the presence of MRS 2179 were similar in control arteries (Fig. 3D).

At 100 μM HOCl, reactive blue 2 (30 μM), a non-selective P2Y receptor antagonist, did not alter the response of HOCl (Fig. 3E). With 500 μM HOCl, the rapid relaxation and subsequent contraction were not different to those observed in the presence of reactive blue 2 compared to the control, in addition, the later relaxation response was similar in the control artery and in the presence of reactive blue 2 (Fig. 3F).

NF449 (10 μM), a P2X1 receptor antagonist, neither altered the transient relaxation nor slow relaxation to 100 μM HOCl compared to the control (Fig. 3G). In addition, there was no difference between responses in the presence of NF449 and the control at 500 μM HOCl (Fig. 3H).

At 100 μM HOCl, α,β-methylene ATP (10 μM), a P2X1 receptor desensitizing agent, did not alter the response in the PCA; both the rapid relaxation and the later relaxation response were similar in the absence and presence of α,β-methylene ATP (Fig. 3I). With 500 μM HOCl, the rapid relaxation, subsequent contraction, and the later relaxation response were not different in the presence of α,β-methylene ATP compared to the control (Fig. 3J).

BX430 (10 μM), a P2X4 receptor antagonist, did not affect the rapid relaxation and the later relaxation evoked by 100 µM HOCl (Fig. 3K). At 500 µM HOCl, the rapid relaxation, subsequent contraction, and later relaxation were not significantly different in the absence and presence of BX430 (Fig. 3L).

These data show that P2X1, P2X4 and P2Y_1_ receptors are not involved in the response to HOCl. Neither PPADS nor reactive blue affected the HOCl responses.

#### Effect of apyrase (hydrolyses nucleotides) and ARL67156 (ecto-ATPase inhibitor)

**Fig. 4:**
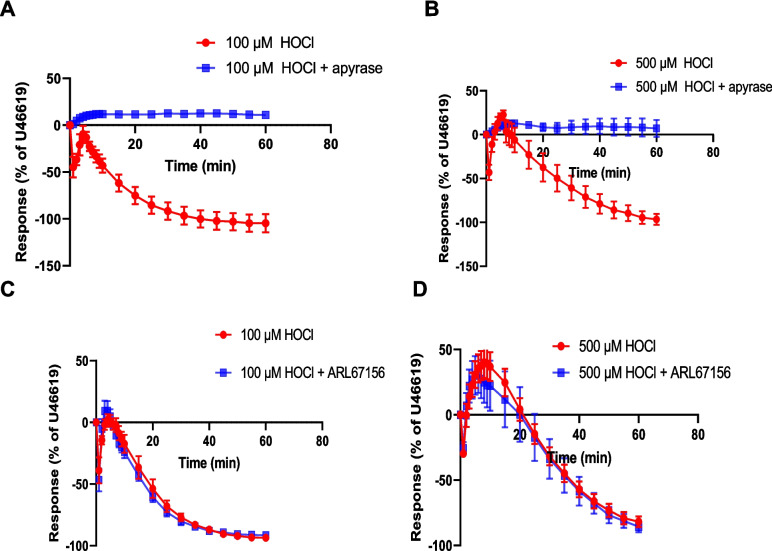
Effect of apyrase (hydrolyses nucleotides) and ARL67156 (ecto-ATPase inhibitor) on responses to HOCl. Responses of porcine coronary arteries to: (**A**) 100 μM HOCl (*n* = 6) and (**B**) 500 μM HOCl (*n* = 6) in the absence or presence of apyrase (100 units/ml). Responses to: (**C**) 100 μM HOCl (*n* = 6) and (**D**) 500 μM HOCl (*n* = 6) in the absence or presence of ARL67156 (100 μM). Artery segments were pre-contracted with U46619. Data are means ± SEM

Apyrase, which hydrolyses nucleotides, was used to investigate the possible involvement of endogenous ATP in the response to HOCl. At 100 μM HOCl, apyrase (100 units/ml) completely blocked the HOCl response compared to the control. The rapid relaxation and slow relaxation in the PCA control group were 58.7 ± 5% and 104.5 ± 9.7%, respectively (*n* = 6), compared to the apyrase group at 4.6 ± 1.3% (*p* < 0.0001) and 10.8 ± 3.4% (*p* < 0.0001) (*n* = 6) (Fig. 4A). Apyrase also abolished the rapid relaxation observed at 500 μM HOCl (0.7 ± 0.5%, *n* = 6) compared to the control group (48.4 ± 5.1%, *n* = 6) (*p* < 0.0001). Moreover, apyrase blocked the contraction produced by 500 µM HOCl (24.9 ± 4.6%, *n* = 6) compared to that in the presence of apyrase (10.7 ± 2.8%, *n* = 6) (*p* = 0.02). In addition, the later relaxation at 60 min to HOCl (96.5 ± 6.2%, *n* = 6) was eliminated in the presence of apyrase (7 ± 9.6%, *n* = 6) (*p* < 0.0001) (Fig. 4B). Apyrase alone elicited vasoconstriction which can be seen in the representative trace in Fig. 7B. A lower concentration of apyrase (10 units/mL) had no significant effect on the relaxation response to HOCl.

ARL67156, an ecto-ATPase inhibitor, was also used to determine whether ATP is involved in any of the components of the HOCl response. ARL67156 (100 μM) did not alter transient or slow relaxation at 60 min of 100 μM HOCl (*p* = 0*.*48) (Fig. 4C). Moreover, there was no difference between responses to 500 μM HOCl in the presence and absence of ARL67156; not in the transient relaxation, the subsequent contraction, and in the later relaxation (Fig. 4D).

#### Effect of ATP on PCA and endothelium involvement

**Fig. 5:**
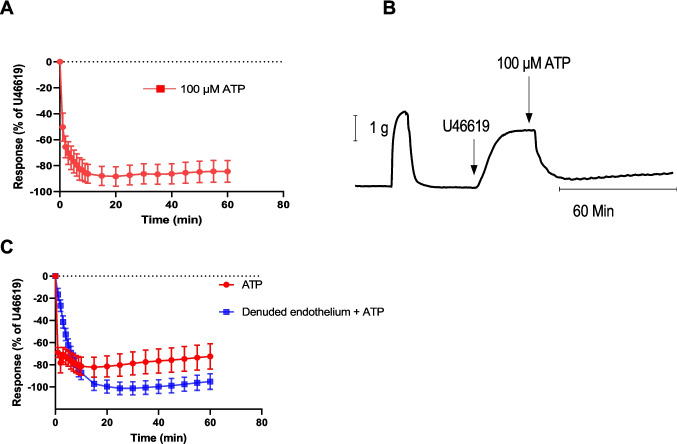
Response to exogenous ATP and effect of endothelium removal. (**A**) Porcine coronary artery (PCA) responses produced by 100 μM ATP (*n* = 8). Artery segments were pre-contracted with U46619. (**B**) Original trace showing the response of the PCA to 100 μM ATP in vessels pre-contracted with U46619; shows the second of two contractions to KCl (60 mM) which was used to set the level of precontraction. (**C**) Responses evoked by 100 μM ATP in endothelium-intact and -denuded PCA (*n* = 8). Data are means ± SEM

The effect of ATP was investigated as a control. ATP was added as a single concentration (100 μM) (rather than as cumulative concentrations) to match the additions of HOCl. ATP caused relaxation in U46619 pre-contracted PCA rings (Fig. 5A). A rapid relaxation response to ATP occurred at 1 min, with a maximum of 50.2 ± 10.7% (*n* = 6). This was followed by a sustained relaxation response with a maximum (at about 10 min) of 88.3 ± 7.1% (*n* = 6). At 60 min, the observed relaxation was 84.4 ± 8.3% (*n* = 6) (Fig. 5A).

The rapid relaxation response of the PCA with intact endothelium to ATP (100 μM) (measured at 1 min) was 68.9 ± 4.3% (*n* = 8). It was blocked after removing the endothelium: 16.5 ± 5.3%, *n* = 8 (*p* < 0.0001). The maximum relaxation response of the PCA with intact endothelium to ATP (100 μM) (measured at ~ 15 min) was 82.2 ± 8.9% (*n* = 6). After removing the endothelium, the relaxation response was larger, being 97.1 ± 6.0% (*n* = 6), but this difference was not statistically significant (*p* = 0.19). At 60 min, the relaxation response in endothelium-denuded PCA was 95.2 ± 7.0% (*n* = 8), which was bigger but not significantly different compared to endothelium-intact PCA, which showed a response of 72.4 ± 11.5% (*n* = 8) (*p* = 0*.*11) (Fig. 5C).

#### Effect of 8-SPT and suramin on ATP response

**Fig. 6:**
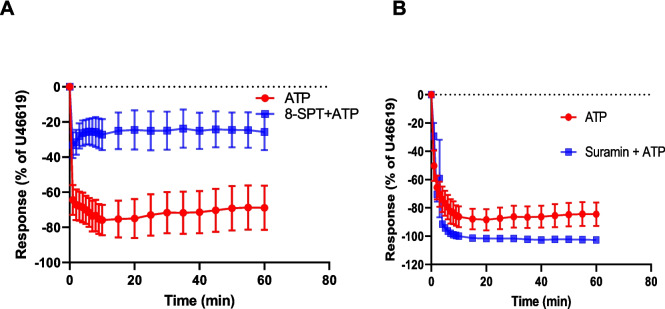
Effect of 8-SPT (adenosine receptor antagonist) and suramin (P2 receptor antagonist and membrane channel inhibitor) on responses to ATP. Porcine coronary artery responses produced by 100 μM ATP in the absence or presence of (**A**) 8-SPT (100 μM) (*n* = 6), and (**B**) suramin (100 μM) (*n* = 6). Artery segments were pre-contracted with U46619. Data are means ± SEM

The effects of 8-SPT and suramin on the response to exogenous ATP were investigated to characterise the purine receptors involved. 8-SPT (100 μM) reduced the rapid relaxation response caused by ATP (100 μM) (33.2 ± 7.43%, *n* = 6) compared to the relaxation response observed under normal conditions (64.3 ± 8.4%, *n* = 6) (*p* = 0.02) (Fig. 6A). It also reduced the maximum relaxation response caused by ATP (100 μM) (36.6 ± 7.8%, *n* = 6) compared to the relaxation response observed under normal conditions (84.1 ± 6.1%, *n* = 6) (*p* = 0.0009) Furthermore, after 60 min, the relaxation response to ATP in the presence of 8-SPT (25.7 ± 10.3%, *n* = 6) was significantly lower than that in the control group (68.8 ± 12.6%, *n* = 8) (*p* = 0.03) (Fig. 6A).

The presence of suramin (100 μM) did not alter the response of PCA to ATP; the maximum relaxation was 87.8 ± 7.3% (*n* = 6) in the absence of suramin compared to 101.3 ± 1.3% (*n* = 6) in the presence of suramin (*p* = 0.09). Likewise, the relaxation responses at 60 min in the absence or presence of suramin were similar at 84.4 ± 8.3% and 102.6 ± 1.6% (*n* = 6) (*p* = 0.05), respectively (Fig. 6B).

Thus, ATP causes relaxation via P1 receptors, but not via suramin-sensitive P2 receptors.

#### Effect of apyrase on ATP and adenosine responses

**Fig. 7:**
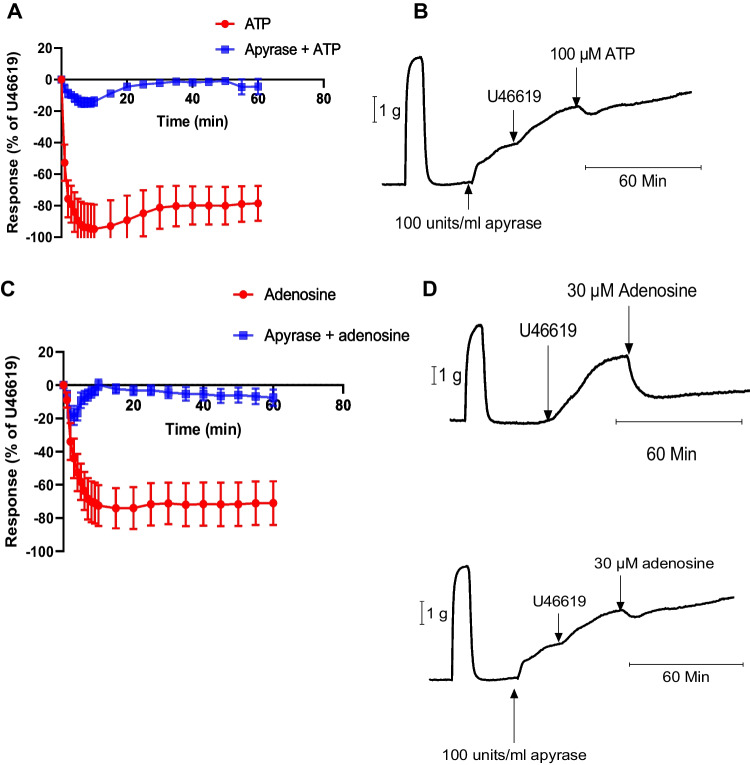
Effect of apyrase on response to ATP and adenosine. (**A**) Responses of porcine coronary arteries (PCA) mediated by 100 μM ATP in the absence or presence of apyrase (100 units/ml) (*n* = 6). (**B**) Original trace showing the responses of PCA to 100 μM ATP in a PCA pre-contracted with U46619 in the presence of apyrase (100 units/ml); shows the second of two contractions to KCl (60 mM) which was used to set the level of precontraction. (**C**) Responses to 30 μM adenosine in the absence and presence of apyrase (100 units/ml). (**D**) Representative traces showing responses to 30 μM adenosine in the absence and presence of apyrase (100 units/ml). Data are means ± SEM

The effect of apyrase, which hydrolyses nucleotides, on the ATP response was investigated. Apyrase (100 units/mL) completely inhibited the maximum ATP response when compared to the control group, with relaxation of 8.8 ± 2.1% and 92.2 ± 1.1% respectively (*p* = 0.0005) (*n* = 6). At 60 min, the control group exhibited a relaxation of 78.5 ± 11.0% (*n* = 6), whereas the apyrase group showed a significantly lower relaxation of 4.4 ± 4.9% (*p* = 0.0001) (Fig. 7A, B). Apyrase (100 units/mL) also blocked the relaxation response to adenosine (30 µM). The adenosine relaxation at 15 min, 74 ± 12.1% (*n* = 6), was completely blocked by 100 units/ml of apyrase, 19.0 ± 4.9% (*n* = 6) (*p* = 001). The relaxation at 60 min in the control group was 70.9 ± 13.1% (*n* = 6) compared to the apyrase group at 7.5 ± 4.9% (n = 6) (*p* = *0.0*01) (Fig. 7C, D). Apyrase (100 units/mL) had no significant effect on relaxation responses to bradykinin: the bradykinin pEC_50_ of 8.5 ± 0.4 and R_max_ = 64.5 ± 4.5% (*n* = 6) were not changed in arteries with added apyrase, with a pEC_50_ of 8.4 ± 0.5 and R_max_ = 61.3 ± 4.8% (*n* = 6). Apyrase alone evoked a vasocontractile effect on the PCA (Fig. 7B, D).

#### Effect of probenecid (pannexin 1 channel blocker) and carbenoxolone (pannexin 1 and connexin channels blocker)

**Fig. 8:**
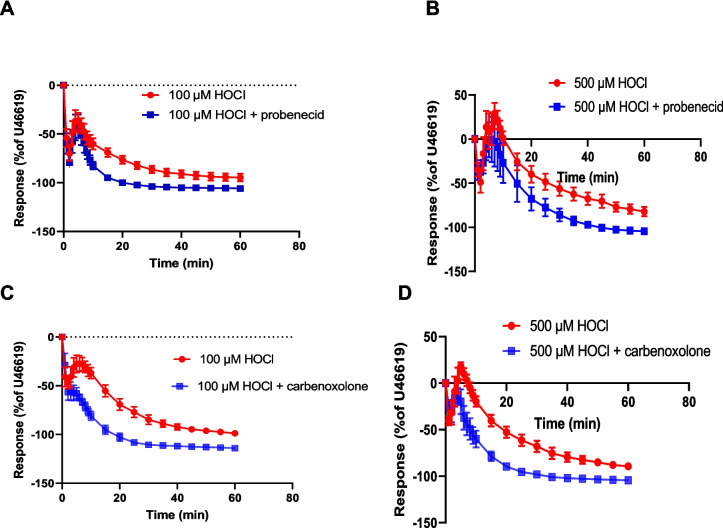
Effect of probenecid and carbenoxelone on responses to HOCl. Porcine coronary artery responses produced by (**A**) 100 μM HOCl (*n* = 7) and (**B**) 500 μM HOCl (*n* = 6) in the absence or presence of probenecid (1 mM), and (**C**) 100 μM HOCl (*n* = 6) and (**D**) 500 μM HOCl (*n* = 6) in the absence or presence of carbenoxolone (100 μM). Artery segments were pre-contracted with U46619. Data are means ± SEM

Suramin can inhibit connexin and pannexin channels [51]. For that reason the pannexin1 and connexin channels blockers probenecid and carbenoxolone were used to investigate whether these channels might be involved in the inhibitory effects of suramin on HOCl responses. Probenecid (1 mM) had no effect on the initial relaxation response to 100 µM HOCl (78.5 ± 2.1%, n = 7) compared to the control (69.7 ± 7.1%, n = 7) (p = 0.25). The later relaxation after 60 min was significantly increased in the presence of probenecid (105.8 ± 1.0%, n = 7) compared to the control group (94.7 ± 3.9%, n = 7) (p = 0.01) (Fig. 8A).

In the presence of probenecid, the initial rapid relaxation to 500 µM HOCl was 47.1 ± 8.1% (*n* = 6) and in the control group it was similar at 52.3 ± 10.8% (*n* = 6) (*p* = 0*.*7). The subsequent contraction to 500 µM HOCl was 29.8 ± 12.1% (*n* = 6) which was significantly reduced in the presence of probenecid 11.1 ± 13.1% (*n* = 6) (*p* = 0.04). The later slow relaxation after 60 min was significantly increased in the presence of probenecid (104.5 ± 1.7%, *n* = 6) compared to HOCl alone (82.1 ± 5.4%, *n* = 6) (*p* = 0.002) (Fig. 8B).

Carbenoxolone (100 μM) failed to block the rapid relaxation evoked by 100 µM HOCl, as is shown in Fig. 8C. On the other hand, the response to HOCl in the presence of carbenoxolone failed to return to baseline (50.2 ± 8.9%, *n* = 6) compared to controls (22.2 ± 7.7%, *n* = 6) (*p* = 0.03). Moreover, HOCl produced a larger relaxation at 60 min in the presence of carbenoxolone compared to HOCl alone (114.1 ± 2.1%, *n* = 6; 98.8 ± 1.6%, *n* = 6, respectively) (*p* = 0.0002) (Fig. 8C).

At 500 µM HOCl alone, the early relaxation was 51.6 ± 5.2% (*n* = 6) in the control, which was not significantly different in the presence of carbenoxolone (41.2 ± 6.1%, *n* = 6) (*p* = 0*.*22). However, subsequent contraction was inhibited in the presence of carbenoxolone (1.7 ± 7.4%, *n* = 6) compared to the control (25.6 ± 5.5%, *n* = 6) (*p* = 0.03). The later relaxation after 60 min significantly increased in the presence of carbenoxolone (104.4 ± 1.3%, *n* = 6) compared to the control group (89.3 ± 2.3%, *n* = 6) (*p* = 0.0002) (Fig. 8D).

#### HOCl releases ATP from cultured human coronary artery endothelial cells

**Fig. 9:**
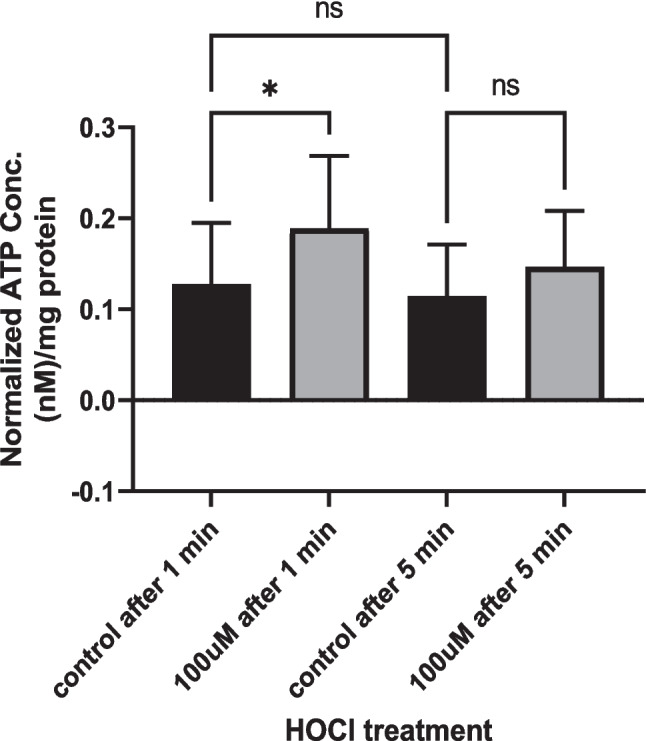
ATP release from cultured human coronary artery endothelial cells (HCAECs) by 100 µM HOCl. The culture medium bathing the cells was assayed for ATP at 1 and 5 min after the addition of HOCl (*n* = 9). *p < 0.05 (two-way ANOVA)

The ATP level in the supernatant of cells with added HOCl (100 µM) at 1 min was 0.18 ± 0.07 nmol/mg protein, which was significantly higher than that in the control group at 0.12 ± 0.06 nmol/mg protein (*n* = 9) (*p* = 0.02) (Fig. 9). The ATP level was not significantly different from the control at 5 min after HOCl addition.

## Discussion

This study has shown that the inflammatory mediator HOCl evokes a multiphasic response in the PCA with components including endothelium-dependent rapid relaxation and contraction, and endothelium-independent sustained relaxation. Use of 8-SPT suggests that the rapid relaxation and contraction evoked by HOCl may be mediated by P1 receptors, implicating an involvement of endogenous adenosine, although neither ZM 241385, an A_2A_ receptor antagonist, nor DPCPX an A_1_ receptor antagonist, were effective. Suramin, a P2 receptor antagonist, also inhibited rapid relaxation and contraction to HOCl, but there was no effect of other P2 receptor antagonists (PPADS, reactive blue 2, NF449, MRS2179, and BX430). Moreover, suramin did not affect relaxation to exogenous ATP, which was blocked by 8-SPT. Thus, the response to HOCl does not appear to involve P2 receptors. Suramin inhibition of HOCl responses could, therefore, be mediated by its inhibition of membrane channels and ATP release from PCA evoked by HOCl, with the released ATP being rapidly metabolised to adenosine leading to activation of P1 receptors. Luminescent ATP assay showed that HOCl elicited ATP release from cultures of human coronary artery endothelial cells.

### Multiphasic response to HOCl

HOCl (100 and 500 μM) evoked a multiphasic response in the PCA which was concentration dependent and involved endothelium-dependent rapid relaxation and contraction, and endothelium-independent sustained relaxation. The concentrations of HOCl used in this study are in line with those used by others (e.g. [35, 52]). Much higher concentrations of HOCl may occur at sites of inflammation following its generation by accumulated neutrophils, with interstitial fluid of inflamed tissues estimated to have concentrations of HOCl of up to 25–50 mM [31]. Relaxation evoked by HOCl could act alongside relaxation produced by other leukocyte-derived inflammatory mediators such as bradykinin and histamine to increase blood flow and delivery of immune cells to the area of inflammation. The contraction to HOCl seen in PCA has also been reported in other tissues. In isolated perfused guinea pig hearts HOCl (1–2 μM) has been shown to cause a reduction in coronary blood flow [53]. HOCl (1 mM to 0.1 M) in sheep pulmonary arteries exhibited contraction under resting force and relaxation in pulmonary arteries pre-contracted with serotonin [54]. Sodium hypochlorite (NaOCl) (75 μM to 5 mM) has been found to mediate endothelium-independent contractions in rat aorta [33].

### Involvement of purine receptors in the response to HOCl

To test our hypothesis that HOCl evokes ATP release from blood vessels, which mediates the vasomotor effects of HOCl, we investigated the possible involvement of P2 receptors in the response to HOCl. P2 receptors expressed in PCA include P2Y_1_ receptors which were shown to mediate increases in coronary blood flow to 2MeSADP and ATP in pigs in vivo which were blocked by MRS2179 (P2Y_1_ receptor antagonist); UTP also caused an increase in coronary blood flow that was not blocked by MRS2179 [55]. PCA also express smooth muscle P2 receptors mediating contraction to UTP which was blocked by suramin but not PPADS [56]. P2Y_14_ receptors mediating contraction to UDP-glucose have been reported in pig and mouse coronary arteries [57, 58]. In the present study suramin, a non-selective P2 receptor antagonist, inhibited rapid relaxation and contraction to HOCl, suggesting a possible involvement of P2 receptors. However, there was no effect of other selective and non-selective P2 receptor antagonists: PPADS, reactive blue 2, NF449 (P2X1 selective), MRS2179 (P2Y_1_ selective), and BX430 (P2X4 selective). Moreover, suramin did not affect the relaxation response to exogenous ATP, which was blocked by 8-SPT. Thus, the rapid vasorelaxant response to HOCl does not appear to involve P2 receptors. An involvement of P2 receptors in the contractile response to HOCl also seems to be excluded because contraction was blocked with 8-SPT.

In the presence of 8-SPT, a P1 receptor antagonist, rapid relaxation and contraction to HOCl were abolished suggesting an involvement of P1 receptors and implicating endogenous adenosine and seeming to rule out an involvement of P2 receptors. Adenosine mediates vasorelaxation of the PCA via A_2A_ receptors, and to a lesser extent A_2B_ receptors, present on the endothelium and smooth muscle [30, 39, 59, 60]. The subtype identity of the P1 receptors mediating contraction to HOCl in the PCA is unclear, but the most likely candidate seems to be A_1_ receptors, which can negatively modulate coronary vasodilatation induced by A_2A_ and A_2B_ receptors [60]. However, neither an A_1_ receptor antagonist, DPCPX, nor an A_2A_ receptor antagonist, ZM 241385, affected any component of the response to HOCl. ZM 241385 has been shown by others to inhibit relaxation responses induced by the adenosine receptor agonists 5′-N-carboxamido adenosine (NECA) and CGS21680 in PCA [43]. The reason for the lack of agreement between our results with 8-SPT, DPCPX and ZM 241385 is unclear and further research is needed.

The prolonged endothelium-independent relaxation to HOCl appears to be independent of both P1 and P2 receptors because it was not blocked by either 8-SPT or suramin or any other of the P2 receptor antagonists. Thus the inhibitory effects of 8-SPT and suramin were selective for the rapid relaxation and contractile components of the HOCl response.

### Involvement of endogenous purines in the response to HOCl

An involvement of P1 receptors in the response to HOCl implicates endogenous adenosine. Exposure of HCAECs to HOCl led to rapid ATP release into the extracellular medium which was detected by luminescence assay. A similar release of ATP by HOCl in PCA, from endothelial and/or smooth muscle cells, could lead to the generation of adenosine following rapid hydrolysis of ATP by E-NTPDases to ADP and AMP, and then to adenosine by 5’-ectonucleotidase [26, 27]. Indeed, PCA relaxation to exogenous ATP was inhibited by 8-SPT, consistent with its rapid metabolism to adenosine and subsequent activation of P1 receptors. HOCl-evoked ATP release could involve pannexin and connexin hemichannels, which could account for the antagonism of HOCl responses by suramin, which can block pannexin and connexin channels and ATP release [12, 51]. Neither probenecid nor carbenoxelone blocked HOCl evoked responses in the PCA so the identity of any membrane channels involved is unclear. It is also possible that HOCl induces release of ATP and adenosine through other mechanisms, for example by formation of membrane protein crosslinks leading to cell lysis [6].

We investigated the effects of apyrase, which hydrolyses nucleotides, and ARL67156, an inhibitor of nucleotide degradation, to further understand the involvement of endogenous purines in the response to HOCl. Apyrase blocked the response to HOCl and exogenous ATP, but it also blocked relaxation to adenosine which was unexpected because apyrase hydrolyses only tri- and di-phosphates. The effective concentration of apyrase, 100 units/ml, was higher than that used previously in platelets and blood vessels (e.g. 0.67, 0.8, 8 and 10 units/ml; [47, 61–63]), although higher concentrations of apyrase (100 and 200 units/ml) have been used in other preparations [48]. A lower concentration of apyrase, 10 units/ml, was ineffective against responses to HOCl and it is possible that HOCl inhibited apyrase activity through amino acid oxidation. Madry et al. have reported that commercially available apyrase solutions contain high concentrations of potassium which caused depolarisation of microglia in rat brain slices and accounted for its effects on microglial ramification and surveillance rather than this being mediated by ATP depletion [48]. It seems likely that our apyrase solution similarly contained potassium and that depolarisation accounted for its inhibition of HOCl, ATP and adenosine responses in the PCA. The fact that apyrase caused vasoconstriction of PCA and that high potassium blocks the response to HOCl (100 µM) (data not shown) is consistent with this. ARL67156 (100 µM) had no effect on responses to HOCl. We have previously shown that ARL67156 (100 µM) enhanced contractions to UTP in porcine coronary artery [56]. Others have reported that ARL67156 (10–100 µM) was unable to suppress the degradation of ATP in mouse colon although it did suppress the degradation of ADP [64]. These results with apyrase and ARL67156 are, therefore, inconclusive regarding the involvement of endogenous ATP as a mediator of the HOCl response in PCA.

## Conclusions

This study aimed to gain a better understanding of vascular inflammation and possible purine receptor involvement in the vascular response produced by HOCl. The study has characterised the multiphasic vasomotor response to HOCl and shown that this may involve activation of P1 receptors, implicating endogenous adenosine released from PCA by HOCl which may come from metabolised ATP released by HOCl. It would be interesting to investigate the role of purinergic signalling in HOCl responses in other blood vessels including small coronary arteries given the heterogeneity of purine receptor expression. The present results may inform about early events involved in ischemic heart disease which is associated with the generation of HOCl and other oxidants by neutrophils. ATP is a chemotaxant so its release by HOCl from blood vessels in vivo could, in addition to effects on vascular contractility, promote the recruitment and activation of circulating immune cells. Thus, our study provides a broader understanding of the potential roles of purines in inflammation.

## Data Availability

No datasets were generated or analysed during the current study.
